# Convolutional Neural Network Can Recognize Drug Resistance of Single Cancer Cells

**DOI:** 10.3390/ijms21093166

**Published:** 2020-04-30

**Authors:** Kiminori Yanagisawa, Masayasu Toratani, Ayumu Asai, Masamitsu Konno, Hirohiko Niioka, Tsunekazu Mizushima, Taroh Satoh, Jun Miyake, Kazuhiko Ogawa, Andrea Vecchione, Yuichiro Doki, Hidetoshi Eguchi, Hideshi Ishii

**Affiliations:** 1 Department of Gastroenterological Surgery, Graduate School of Medicine, Osaka University, 2-2 Yamadaoka, Suita, Osaka 565-0871, Japan; kyanagisawa@gesurg.med.osaka-u.ac.jp (K.Y.); aasai@cfs.med.osaka-u.ac.jp (A.A.); tmizushima@gesurg.med.osaka-u.ac.jp (T.M.); ydoki@gesurg.med.osaka-u.ac.jp (Y.D.); heguchi@gesurg.med.osaka-u.ac.jp (H.E.); 2 Department of Medical Data Science, Graduate School of Medicine, Osaka University, 2-2 Yamadaoka, Suita, Osaka 565-0871, Japan; mkonno@cfs.med.osaka-u.ac.jp; 3 Department of Frontier Science for Cancer and Chemotherapy, Graduate School of Medicine, Osaka University, Osaka 565-0871, Japan; tsatoh@gesurg.med.osaka-u.ac.jp; 4 Department of Radiation Oncology, Graduate School of Medicine, Osaka University, 2-2 Yamadaoka, Suita, Osaka 565-0871, Japan; m_toratani@radonc.med.osaka-u.ac.jp (M.T.); kogawa@radonc.med.osaka-u.ac.jp (K.O.); 5 Department of Radiation Oncology, Osaka International Cancer Institute, 3-1-69 Otemae, Chuo-ku, Osaka-shi, Osaka 541-8567, Japan; 6 Artificial Intelligence Research Center, The Institute of Scientific and Industrial Research, Osaka University, 8-1 Mihogaoka, Ibaraki, Osaka 567-0047, Japan; 7 Institute for Datability Science, Osaka University, 2-8 Yamadaoka, Suita, Osaka 565-0871, Japan; niioka@ids.osaka-u.ac.jp; 8 Global Center for Medical Engineering and Informatics, Osaka University, 1-3 Yamadaoka, Suita, Osaka 565-0871, Japan; jun_miyake@bpe.es.osaka-u.ac.jp; 9 Department of Clinical and Molecular Medicine, University of Rome “Sapienza”, Santo Andrea Hospital, via di Grottarossa, 1035-00189 Rome, Italy; andrea.vecchione@uniroma1.it

**Keywords:** deep learning, convolutional neural network, single cancer cell, chemotherapy, resistance

## Abstract

It is known that single or isolated tumor cells enter cancer patients’ circulatory systems. These circulating tumor cells (CTCs) are thought to be an effective tool for diagnosing cancer malignancy. However, handling CTC samples and evaluating CTC sequence analysis results are challenging. Recently, the convolutional neural network (CNN) model, a type of deep learning model, has been increasingly adopted for medical image analyses. However, it is controversial whether cell characteristics can be identified at the single-cell level by using machine learning methods. This study intends to verify whether an AI system could classify the sensitivity of anticancer drugs, based on cell morphology during culture. We constructed a CNN based on the VGG16 model that could predict the efficiency of antitumor drugs at the single-cell level. The machine learning revealed that our model could identify the effects of antitumor drugs with ~0.80 accuracies. Our results show that, in the future, realizing precision medicine to identify effective antitumor drugs for individual patients may be possible by extracting CTCs from blood and performing classification by using an AI system.

## 1. Introduction

The anticancer chemotherapy is an important first-line treatment in unresectable advanced tumors, such as colorectal cancer [1]. In the cases of colorectal cancer, if the chemotherapy produces a therapeutic effect, subsequent conversion therapy allows R0 resection, and a long-term prognosis can be expected [2]. However, if first-line chemotherapy is not effective, patients will miss valuable treatment opportunities. In the current treatment of colorectal cancer, the evaluation of *EGFR* and *RAS* expression from colorectal cancer resected specimens and biopsy specimens selects anticancer drugs and molecular targeted drugs that can be expected to have therapeutic effects [1].

The ability to predict the effect of an anticancer drug by liquid biopsy would be useful, because it is a minimally invasive procedure. Circulating tumor cells (CTCs) can be used as a type of liquid biopsy [3]. Recently, various methods have been developed for recovering CTCs, and their accuracy is improving [4]. Many studies that focus on sequence analysis of CTCs to examine gene mutations exist [5,6]. However, CTC sequence analysis obtained a large amount of data, making the analysis time-consuming.

Conversely, computer-based analyses of large volumes of data recently have become widely used, as computer performance has improved [7,8]. Many analytical methods, such as statistical analysis, model analysis, simulation analysis and theoretical analysis, have emerged. These computational methods have been successful in many research areas, and numerous machine learning research projects have been reported in recent years [9,10,11]. 

Techniques using AI trained on convolutional neural networks (CNNs) that mimic optical neural networks have recently been developed. To use it in medical applications, there have been various attempts to train AI on medical imaging. Esteva et al. made the first report on the analysis of clinical information using AI [12]. They trained CNN using 129,450 clinical images of 2032 different diseases. Surprisingly, CNN’s performance was comparable to the level of the diagnosis of dermatologists in classifying skin cancer. 

In the field of cancer research, CNN, one of the deep learning algorithms, is rapidly being adopted for analyzing medical images [11]. Therefore, we applied artificial intelligence-based image recognition technology and researched whether it would be possible to simply evaluate anticancer drug resistance from cancer cell morphology, using CNN. In this study, we have constructed the recognition system of single-cell level characters that can be adapted for examining circulating tumor cells, using the deep learning method. As the first step of this strategy, the character of drug-resistance of colorectal carcinoma cell lines to antitumor drugs, 5-fluorouracil (5-FU) and trifluorothymidine (FTD), was determined.

## 2. Results

### 2.1. Models Constructed to Discriminate Resistance and Non-Resistance of Cancer Cells to Anticancer Drugs in the Confluent Category

**Figure 1:**
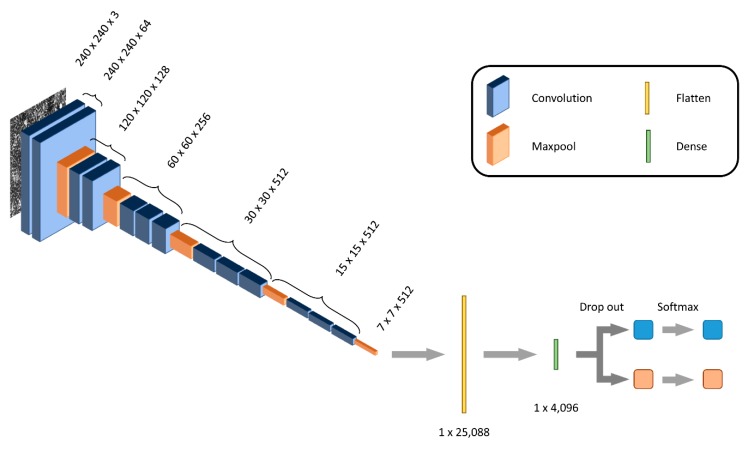
Schematic diagram of our machine learning using the VGG16 model. The VGG16 has 13 convolutional layers, 5 max pooling layers, and 3 connected layers, with a planarization layer and a high-density layer. In this neural network system, input image data could be categorized into two classes, namely, resistant and non-resistant to anticancer drugs.

**Figure 2:**
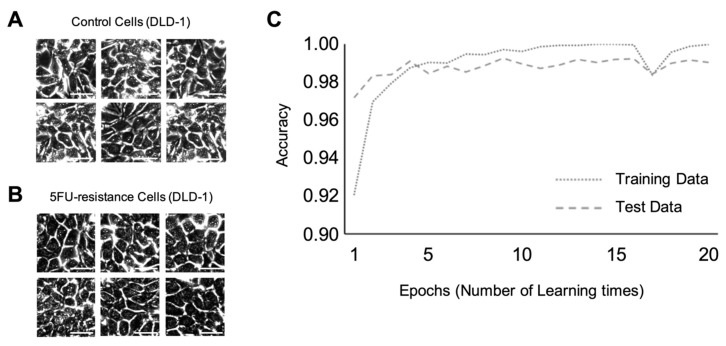
Machine learning at confluence level for DLD-1 cells resistant and non-resistant to anticancer drugs. Scale bar; 50 µm (**A**) Representative input image of the control DLD-1 cells. Scale bar; 50 µm (**B**) Representative input image of anticancer drug-resistant DLD-1 cells. (**C**) Accuracy variation per epoch.

**Figure 3:**
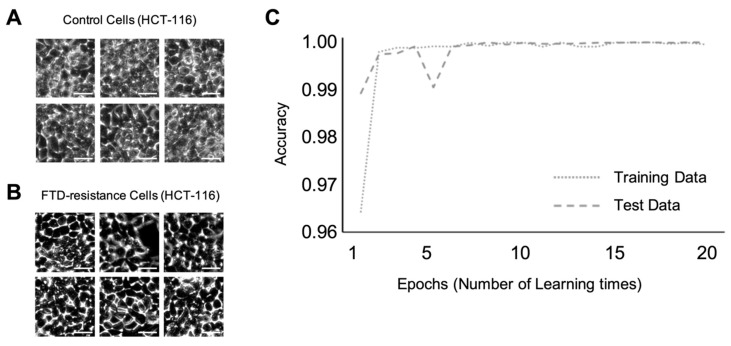
Machine learning at confluence level for HCT-116 cells resistant and non-resistant to anticancer drugs. Scale bar; 50 µm (**A**) Representative input image of the control HCT-116 cells. Scale bar; 50 µm (**B**) Representative input image of drug-resistant HCT-116 cells. (**C**) Accuracy variation per epoch.

Using two types of colorectal cancer cell lines, DLD-1 and HCT-116, we constructed the discriminant model using the VGG16 deep learning process (Figure 1). We used a model based on VGG16, and trained the VGG16 model with each of 7500 images of cancer cells that displayed different levels of resistance to anticancer drugs. For this study, we trained only the last three convolutional layers and three connected layers with the selected images. Machine learning was performed for discrimination at the confluence level of cell culture. Figure 2A,B shows the representative input images of the control DLD-1 cells and those resistant to 5-FU anticancer drugs, respectively. The accuracy variation per epoch is shown in Figure 2C. In this figure, the dotted line indicates the accuracy rate on the training data during the learning steps, and the dashed line indicates the accuracy rate on the test data during the validation steps. As shown in this figure, VGG16 trained in discriminant mode could determine which cell class was resistant or non-resistant to anticancer drugs, with an accuracy score of ~0.98. This process was replicated, and machine learning for discriminating which HCT-116 cells were resistant or non-resistant to anticancer drugs was performed. The representative input images of the control and FTD resistant HCT-116 cells are shown in Figure 3A,B, respectively. The time-course variation of accuracy is shown in Figure 3C. The dotted and dashed lines have the same meanings as above. The accuracy rate is higher than in the case of DLD-1 cells. This figure shows that the accuracy rate converges to almost 1.00.

### 2.2. Discrimination Model of the Single-Cell Level

**Figure 4:**
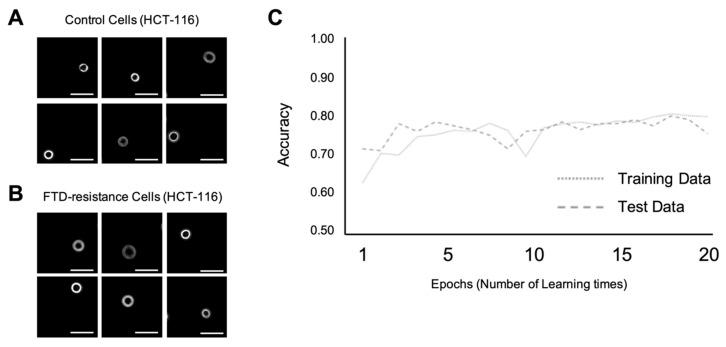
Machine learning at single-cell level for HCT-116 cell resistant and non-resistant to anticancer drugs. Scale bar; 50 µm (**A**) Representative input images of the control HCT-116 cell. Scale bar; 50 µm (**B**) Representative input images of the drug-resistant HCT-116 cell. (**C**) Accuracy variation per epoch.

To use CTC for diagnosis in the future, we attempted discrimination at the single-cell level. In this step, we attempted to construct a discrimination model with machine learning using HCT-116 cell images that are either resistant or non-resistant to FTD anticancer drugs. As in the case of the confluence level training described above, Figure 4A,B shows the representative input of 1000 images of a control cell and a cell resistant to FTD anticancer drugs. Figure 4C indicates the time-course variation of the accuracy rate. The dotted and dashed lines have the same representation as above. The accuracy rate of the discrimination model using the training data and test data increased to 0.7–0.8, respectively, as the number of epochs increased; the sensitivity was 0.68, the specificity was 0.76, and the accuracy was 0.72, at the 20th epochs.

## 3. Discussion

Conventionally, in many reports on cancer cell imaging, much effort has been put into classifying cell populations. However, in this study, for the first time, machine learning could discriminate the characteristics of cancer cells, even at the single-cell level. In this study, it was possible to classify cell populations according to their characteristics with more than 0.98 accuracies, using the modified VGG16 neural network model, as shown in Figure 2 and Figure 3. This is an improvement on the results from previous studies. Our model could discriminate cell characteristics with 0.7–0.8 accuracies even at the single-cell level. It is more difficult to discriminate for single cells by the CNN image recognition system than to do so for a cell population; however, the result demonstrates that single-cell discrimination may be possible at an acceptable level. As previously mentioned, we employed the DLD-1 cell line to determine whether it was resistant to 5-FU. HCT-116 was employed to determine whether it was resistant to FTD. Below are some of the reasons why the resistance level of DLD-1 to 5-FU is lower than that of HCT-116, and the resistance level to FTD of HCT-116 is lower than that of DLD-1. For machine learning at the single-cell level, we selected cases of FTD resistance in HCT-116. When comparing the degree of resistance, the ratio of the IC_50_ value between the control and resistant cells, HCT-116 in FTD resistance, was 31.1 μM, whereas DLD-1 in 5-FU resistance exceeded an estimated 80.0 μM (data not shown). This is the reason why we selected the HCT-116 cell line for machine learning at the single-cell level. 

**Figure 5:**
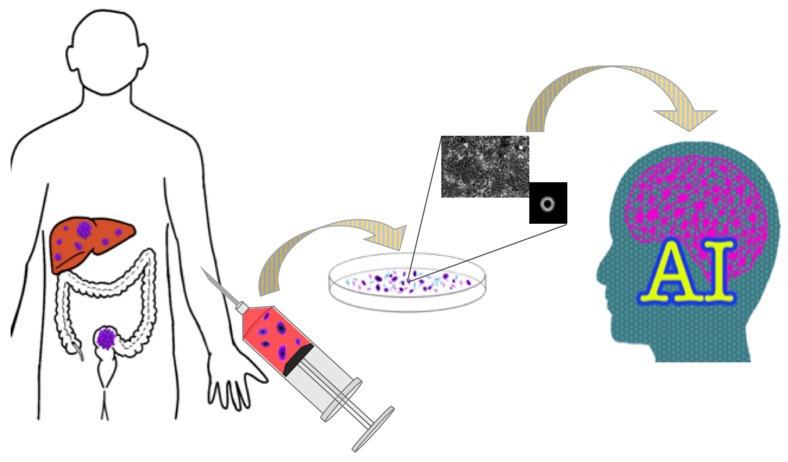
Schematic diagram of colorectal cancer precision medicine using AI. Based on the circulating tumor cell (CTC) morphology detected in the liquid biopsies of patients with unresectable advanced colorectal cancer, the presence or absence of anticancer drug resistance is determined by image recognition technology, using deep learning.

Considering that our model maintained ~0.80 accuracies, even with a cell line having such a low level of resistance, it is no exaggeration to say that we have established a foundation that is a great step forward in devising a single-cell-level character recognition system that is adaptable for examining circulating tumor cells. Figure 5 shows a future ideal model of precision medicine that predicts the effect of an anticancer drug using AI analysis of CTCs. A blood sample would be collected from the patient with multiple metastatic tumors, and CTCs would be extracted. The AI analysis of CTCs would be able to predict the effective anticancer drug for the patient and is expected to construct the optimal treatment strategy for the patient. Our results advance predictive medicine, including the prediction of treatment effects, and contribute to the realization of personalized medicine.

## 4. Materials and Methods

### 4.1. Cell Lines and Cell Culture

In this study, we used human colorectal carcinoma cell lines, HCT-116 and DLD-1, as controls. These were purchased from the American Type Culture Collection (Manassas, VA, USA) and maintained in Dulbecco’s Modified Eagle’s Medium (Sigma-Aldrich, St. Louis, MO, USA) supplemented with 10% FBS at 37 °C and 5% CO_2_ in a humidified incubator. In our previous study [13] on resistance to anticancer drugs, cell lines for 5-FU and FTD were established for the machine learning process. We also used the HCT-116 cell line, which is characteristically FTD resistant, and the DLD-1 cell line, which is 5-FU resistant. The resistance level of HCT-116 to FTD is lower than that of DLD-1. However, the resistance level of DLD-1 to 5-FU is lower than that of HCT-116. To establish the single-cell level character recognition system that is adaptable for examining circulating tumor cells, the system must be able to determine whether there is resistance to anticancer drugs or not, even if the resistance difference, when compared with control cells, is not large. This was the reason for selecting these anticancer drug-resistant cell lines. 

### 4.2. Cytotoxicity Assay

The cell lines were seeded at a density of 4 × 10^3^ cells per well in 96-well plates and then pre-cultured for 24 h. They were exposed to various concentrations of FTD and 5-FU antitumor drugs, for 72 h. The in vitro cytotoxic effects were assayed using the Cell Counting Kit-8 (Dojindo, Tokyo, Japan).

### 4.3. Preparation of Image for Deep Learning

The phase-contrast images of the colorectal cell lines, HCT-116 and DLD-1, were obtained with a microscope (B-X700, KEYENCE). The machine learning datasets were comprised of two categories with 9000 images of cell confluence and 1100 images of single cells. The images in each dataset were 240 × 240 pixels in size. The confluence category training dataset had 7500 images and its test set had 1500 images. For the single-cell dataset, 1000 images were used as the training set, and 100 images were used as the test set. During preparation, the samples were all converted to gray-scale images.

### 4.4. The Machine Learning Process with a Neural Network System

A convolutional neural network (CNN) is a machine learning model, which is a system of convolutional, pooling layers and fully connected layers [14,15]. The convolutional layers detect local features in the input data, whereas the pooling layers reduce the computational load as well as the risks of overfitting and image shift. VGG16 is one of the CNN model systems and is pre-trained to classify 1.2 million images into 1000 categories. Many target classes can be classified easily using this VGG16 model even without pre-learning the 1000 categories. We, therefore, trained the VGG16 model (as shown in Figure 1) with images of cancer cells that displayed different levels of resistance to anticancer drugs. For this study, we trained only the last three convolutional layers and three connected layers with the selected images. For testing our model and to validate the training, we used Google’s TensorFlow [14] deep learning framework, and Keras [16,17] using TensorFlow backend.
